# Comparison of cephalometric measurements between conventional and automatic cephalometric analysis using convolutional neural network

**DOI:** 10.1186/s40510-021-00358-4

**Published:** 2021-05-31

**Authors:** Sangmin Jeon, Kyungmin Clara Lee

**Affiliations:** 1 Gwangju, Republic of Korea; 2 Department of Orthodontics, School of Dentistry, Chonnam National University, 33 Yongbong-ro, Buk-gu, Gwangju, 61186 Republic of Korea

**Keywords:** Cephalometric analysis, Artificial intelligence, Machine learning, Convolutional neural network

## Abstract

**Objective:**

The rapid development of artificial intelligence technologies for medical imaging has recently enabled automatic identification of anatomical landmarks on radiographs. The purpose of this study was to compare the results of an automatic cephalometric analysis using convolutional neural network with those obtained by a conventional cephalometric approach.

**Material and methods:**

Cephalometric measurements of lateral cephalograms from 35 patients were obtained using an automatic program and a conventional program. Fifteen skeletal cephalometric measurements, nine dental cephalometric measurements, and two soft tissue cephalometric measurements obtained by the two methods were compared using paired *t* test and Bland-Altman plots.

**Results:**

A comparison between the measurements from the automatic and conventional cephalometric analyses in terms of the paired *t* test confirmed that the saddle angle, linear measurements of maxillary incisor to NA line, and mandibular incisor to NB line showed statistically significant differences. All measurements were within the limits of agreement based on the Bland-Altman plots. The widths of limits of agreement were wider in dental measurements than those in the skeletal measurements.

**Conclusions:**

Automatic cephalometric analyses based on convolutional neural network may offer clinically acceptable diagnostic performance. Careful consideration and additional manual adjustment are needed for dental measurements regarding tooth structures for higher accuracy and better performance.

## Introduction

Cephalometric analysis is an essential diagnostic tool for the treatment planning and evaluation of orthodontic patients. Accurate identification of the anatomical landmarks on cephalograms is critical for a reliable cephalometric analysis [1]. Lateral cephalometric radiographs have been employed as an essential tool in orthodontics. However, to analyze such radiographs, the important anatomical structures need to be identified by a landmark identification and manual tracing process. However, this analysis requires a skilled orthodontist, and the process is time-consuming.

In computer science, artificial intelligence (AI) refers to the study of systems that perform tasks that require human intelligence using different computerized algorithms [2, 3]. Machine learning is a method of data analysis that allows computer programs to automatically improve through cognitive content. It is a branch of technology that allows systems to learn from data, identify patterns, and make decisions with minimal human intervention [4]. These programs make decisions by examining large amounts of input data and with known outputs, subsequently, drawing conclusions on the input data with unknown outputs based on the initial “training” process.

In recent years, the use of AI in medicine and healthcare for the diagnosis and treatment of patients has been a topic of significant interest [5]. This has resulted in the application of AI and machine learning technologies to dental processes including the classification of temporomandibular joint osteoarthritis and osteoporosis, prediction of the debonding probability of computer-aided design/computer-aided manufacturing (CAD/CAM) crowns, automatic detection and classification of jaw lesions and periodontal bone loss, survival prediction of oral cancer patients, tooth labeling, detection and diagnosis of dental caries, and detection of osteoporosis [6–13]. Furthermore, programs have been developed to automatically digitize the anatomical structures on lateral cephalometric radiographs. With these programs, automatic cephalometric analysis including diagnostic and analytical imaging tasks can be performed by AI and machine learning technologies. However, to the best of our knowledge, few recent studies about AI performance of cephalometric analysis which is useful for clinicians are available. Previous studies about deep learning algorithm reported that AI accurately detected cephalometric landmarks [14, 15]. In order to further explore the application of these technologies to clinical orthodontics, the results of clinical performance of cephalometric analysis are necessary. The purpose of the present study was to evaluate the accuracy of automatic cephalometric analysis by comparing with that of conventional cephalometric measurements.

## Material and methods

**Fig. 1:**
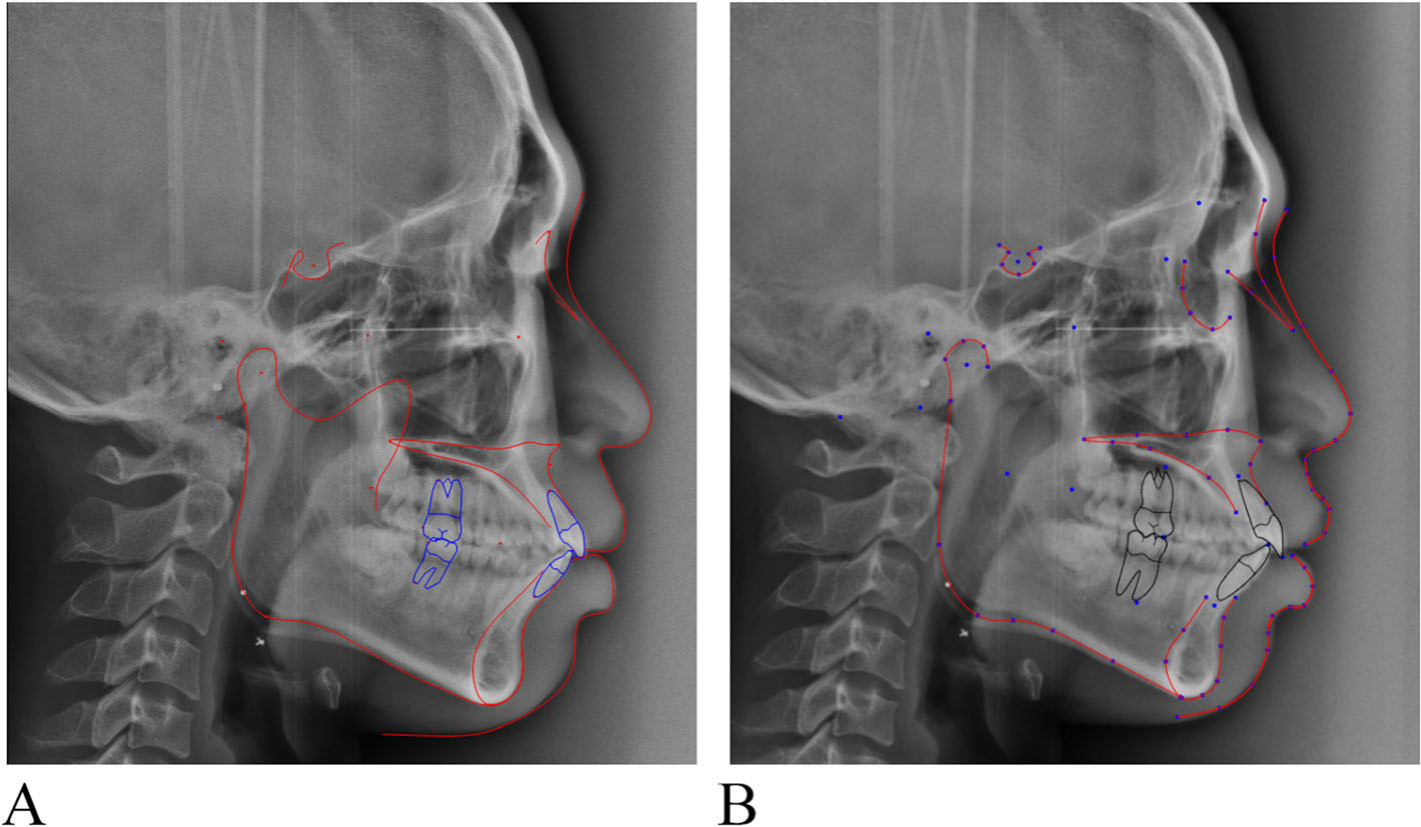
Cephalometric analysis using conventional (**a**) and AI (**b**) methods

This retrospective study was approved by the Institutional Review Board of the Chonnam National University Dental Hospital, Gwangju, Korea (CNUDH-EXP-2019-023). The inclusion criteria were (1) a fully erupted permanent dentition, and (2) no broad prosthetic restorations such as metal crowns or bridges, on the molars. The exclusion criteria were (1) multiple missing tooth and broad prosthetic restorations such as metal crowns or bridges, on the molars and (2) history of orthodontic treatment or orthognathic surgery. Conventional lateral cephalograms of 35 orthodontic patients (20 men, 15 women; mean age = 23.8 years) were obtained using OrthoCeph**®** OC100 (Instrumentarium Imaging Co., Tuusula, Finland). The cephalograms were imported to the V-ceph^TM^ (version 8.0, Cybermed Inc., Seoul, Korea) for the conventional cephalometric analysis and to the CephX^TM^ (ORCA Dental AI Inc., Herzliya, Israel) for the AI analysis (Fig. 1). Sixteen anatomical landmarks were chosen (Table 1), and 15 skeletal cephalometric measurements, 9 dental cephalometric measurements, and 2 soft tissue cephalometric measurements were obtained by an experienced single examiner with over 7 years of experience in orthodontic treatment.

**Table 1:** Description of cephalometric landmarks used in this study

Landmark (abbreviation)	Definition
Sella (S)	Center of the pituitary fossa of the sphenoid bone
Nasion (Na)	Most anterior point on the frontonasal suture in the midsagittal plane
Porion (Po)	Most superior point of the external auditory meatus
Orbitale (Or)	Most inferior point on infraorbital rim
Articulare (Ar)	Point at the junction of the posterior border of the ramus and the inferior border of the posterior cranial base
Posterior nasal spine (PNS)	Posterior spine of the palatine bone constituting the hard palate
Anterior nasal spine (ANS)	Anterior tip of the sharp bony process of the maxilla at the lower margin of the anterior nasal opening
Point A (A)	Deepest point of the curve of the anterior border of the maxilla
Upper incisor (U1)	Tip of the crown of the most anterior maxillary central incisor
Upper first molar (U6)	Most distal point on the crown of upper first molar
Lower incisor (L1)	Tip of the crown of the most anterior mandibular central incisor
Lower first molar (L6)	Most distal point on the crown of lower first molar
Gonion (Go)	Point along angle of the mandible, midway between lower border of mandible and posterior ascending ramus
Point B (B)	Most posterior point in the concavity along anterior border of the symphysis
Pogonion (Pog)	Most anterior point on the midsagittal symphysis
Menton (Me)	Most inferior point of the symphysis

### Statistical analysis

The sample size calculation was performed according to the result of previous study of Hwang et al. [15]. The effect size was calculated to 0.49. A statistical power of 80 percent and a type I error of 5 percent was assumed by the G*power program (version 3.1.9.2, Heinrich-Heine-University, Dusseldorf, Germany). The calculation indicated that 35 individuals were required in the study.

All data were revealed to be normally distributed. Paired *t* test was then performed to determine the differences between the AI and conventional programs. For the purpose of comparing the two measurements obtained from each two methods graphically, the differences between the two methods were plotted using Bland-Altman analysis [16]. Shapiro-Wilk test and paired *t* test were conducted using SPSS software package (version 23.0; IBM, Armonk, NY) and Bland-Altman plots were made by MedCalc (Ostend, Belgium). Significance level was set of 5%. To assess the errors of each method, the process of acquiring measurements using the conventional program was repeated after 2 weeks, and the measurement errors were calculated using Dahlberg’s method [17]. The range of error was 0.1 to 0.3 mm for the linear measurements and from 0.1 to 0.3° for the angular measurements. For inter-examiner reproducibility, the second examiner performed the process of acquiring measurements using the conventional program, and the measurements were compared with first examiner’s measurements using the intraclass correlation coefficient (ICC). The ICC values were found to be statistically insignificant showing a mean of 0.91 (ICC 0.88-0.94), indicated excellent reliability.

## Results

Table 2 summarizes the differences between the measurements obtained by the conventional and AI methods. Statistically significant differences were found in saddle angle, linear measurements of maxillary incisor to NA line, and mandibular incisor to NB line. The soft tissue measurements did not show any significant difference between the two methods.

**Table 2:** Comparison of the cephalometric measurements between conventional and artificial intelligence methods

	Conventional		AI		Difference		Significance (*p* value)
	Mean	SD	Mean	SD	Mean	SD	
**Skeletal measurement**							
Saddle angle (°)	124.6	5.0	123.4	4.7	1.2	2.0	0.002^a^
Articular angle (°)	149.3	6.1	149.9	5.3	−0.6	3.5	0.316
Gonion angle (°)	121.4	5.8	121.9	7.6	−0.5	4.1	0.482
Sum	395.3	6.3	395.2	6.4	0.1	2.7	0.894
Ant. cranial base (mm)	69.5	3.7	68.4	4.5	1.1	2.9	0.228
Post. cranial base (mm)	38.6	4.6	37.1	5.4	1.4	3.6	0.119
Ramus height (mm)	50.4	6.4	49.8	6.7	0.7	3.4	0.257
SNA (°)	80.7	3.8	81.2	3.8	−0.5	1.8	0.114
SNB (°)	77.6	4.5	78.0	4.8	−0.4	1.5	0.143
ANB (°)	3.1	3.2	3.2	3.3	−0.1	1.4	0.618
Facial angle (Down’s)	87.2	3.2	88.0	3.8	−0.8	1.9	0.071
Post.FH/Ant.FH	66.2	5.1	66.1	4.8	0.2	2.5	0.661
Mn. plane angle (°)	35.3	6.3	35.6	6.2	−0.3	2.8	0.511
FMA (°)	26.3	5.7	25.7	6.0	0.6	2.8	0.198
Palatal plane angle (°)	24.6	5.9	24.9	5.7	−0.3	1.7	0.513
**Dental measurement**							
Wits appraisal	−1.3	7.2	−2.5	4.7	1.2	6.7	0.305
FMIA (°)	58.9	8.5	60.0	8.5	−1.1	4.4	0.138
IMPA (°)	95.0	8.3	93.9	7.4	1.1	3.9	0.111
Mx 1 to SN (°)	107.0	11.0	107.6	8.8	−0.6	5.1	0.523
Interincisal angle (°)	122.9	14.8	122.9	11.8	0.1	6.5	0.988
Mx1 to NA (°)	26.3	9.6	26.4	7.2	−0.1	4.6	0.910
Mx1 to NA (mm)	7.5	3.6	5.6	2.6	1.8	2.3	0.001^a^
Mn1 to NB (°)	27.7	7.4	27.6	6.9	0.1	3.6	0.922
Mn1 to NB (mm)	8.3	3.3	6.1	2.8	2.1	1.4	0.000^a^
**Soft tissue measurement**							
Esthetic line to upper lip (mm)	0.6	3.2	0.6	2.9	0.0	1.1	0.899
Esthetic line to lower lip (mm)	1.6	3.4	1.7	3.1	0.0	1.5	0.884

*SD* Standard deviation

^a^The results of paired *t* test

**Fig. 2:**
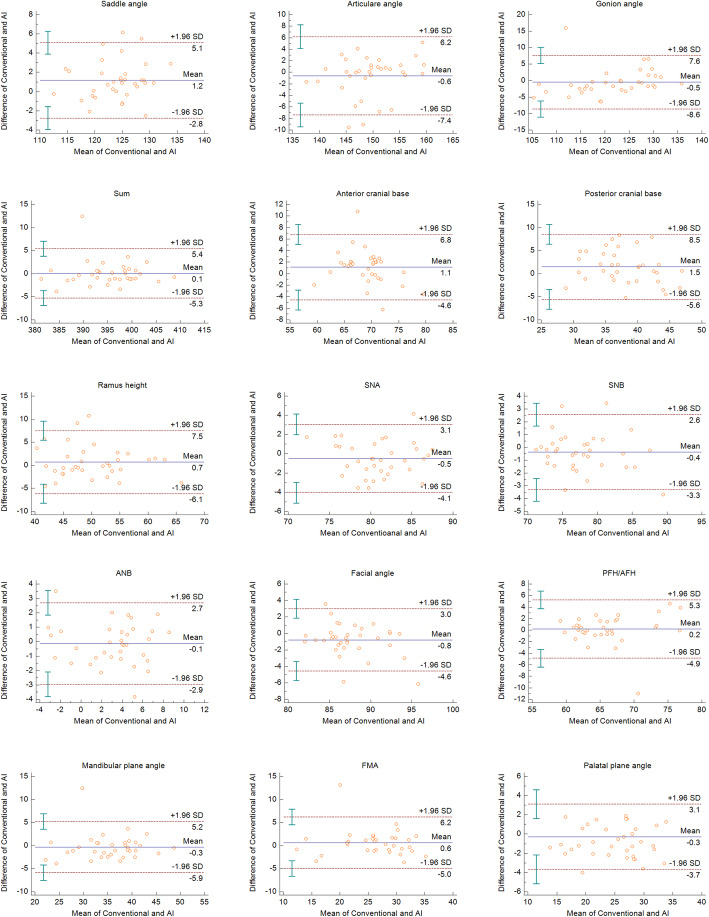
Bland-Altman plots for the skeletal measurements in each conventional and AI methods. For each plot, the *x*-axis represents the mean of the compared measurements, and the *y*-axis represents the difference between the compared measurements. The blue line represents the bias, and the red-hashed lines represent the upper and lower limits of agreement

**Fig. 3:**
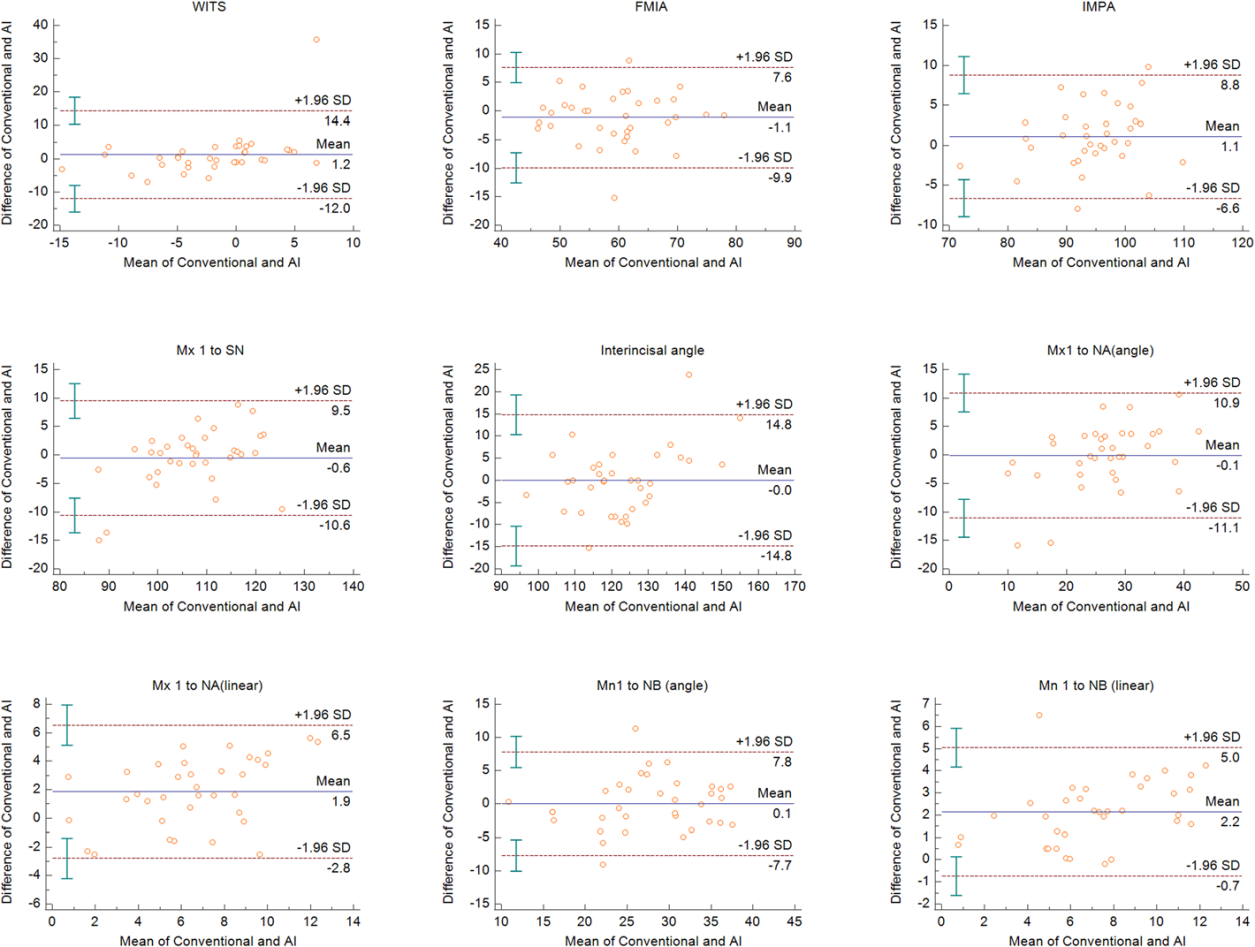
Bland-Altman plots for the dental measurements in each conventional and AI methods

**Fig. 4:**
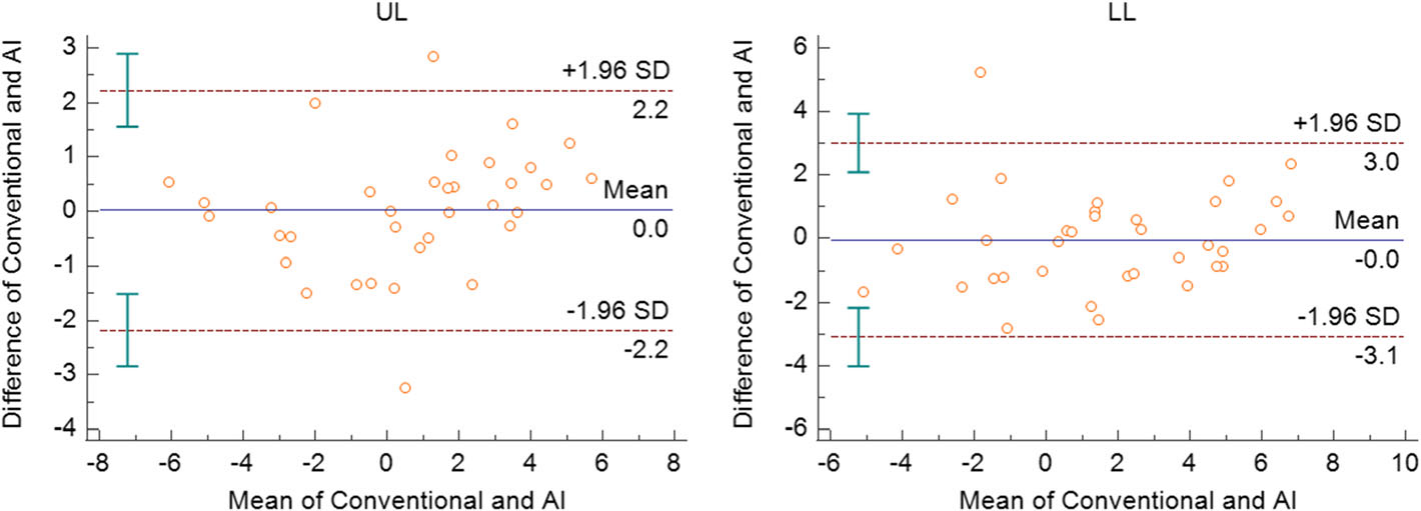
Bland-Altman plots for the soft tissue measurements in each conventional and AI methods

All measurements were within the limits of agreement based on the Bland-Altman plots. The measurements that showed significance in the paired *t* test were within the limits of agreement (Figs. 2, 3, and 4). The widths of limits of agreement were wider in dental measurements than those in the skeletal measurements (Table 3).

**Table 3:** Bland-Altman analysis for the cephalometric measurements between conventional and artificial intelligence methods

	95% of limit of agreement		Width
	Upper limit	Lower limit	
**Skeletal measurement**			
Saddle angle (°)	5.1	−2.8	7.9
Articular angle (°)	6.2	−7.4	13.6
Gonion angle (°)	7.6	−8.6	16.2
Sum	5.4	−5.3	10.7
Ant. cranial base (mm)	6.8	−4.6	11.4
Post. cranial base (mm)	8.5	−5.6	14.1
Ramus height (mm)	7.5	−6.1	13.6
SNA (°)	3.1	−4.1	7.2
SNB (°)	2.6	−3.3	5.9
ANB (°)	2.7	−2.9	5.6
Facial angle (Down’s)	3.0	−4.6	7.6
Post.FH/Ant.FH	5.3	−4.9	10.2
Mn. plane angle (°)	5.2	−5.9	11.1
FMA (°)	6.2	−5.0	11.2
Palatal plane angle (°)	3.1	−3.7	6.8
**Dental measurement**			
Wits appraisal	14.4	−12.0	26.4
FMIA (°)	7.6	−9.9	17.5
IMPA (°)	8.8	−6.6	15.4
Mx 1 to SN (°)	9.5	−10.6	20.1
Interincisal angle (°)	14.8	−14.8	29.6
Mx1 to NA (°)	10.9	−11.1	22.0
Mx1 to NA (mm)	6.5	−2.8	9.3
Mn1 to NB (°)	7.8	−7.7	15.5
Mn1 to NB (mm)	5.0	−0.7	5.7
**Soft tissue measurement**			
Esthetic line to upper lip (mm)	2.2	−2.2	4.4
Esthetic line to lower lip (mm)	3.0	−3.1	6.1

All measurements were within the limit of agreement

## Discussion

In orthodontics, cephalometric analysis is commonly performed by computerized method, which includes manual identification of the landmarks on a monitor. The software automatically calculates the distances and angles which are necessary for the cephalometric analysis. Otherwise, direct tracing of the radiograph is transferred to a computer. These computerized cephalometric analyses may cause some errors, such as transferring and measurement errors, even though the manual landmark identification is performed by a clinician [18, 19]. Leonardi et al. [20] reported that the accuracy of a cephalometric analysis varies between 60 and 80% for a computerized analysis compared with the fully manual process, where the total errors should be no more than 0.59 mm in the *x* direction and 0.56 mm in the *y* direction to be considered acceptable. Recent studies showed that despite this, cephalometric analysis performed by computerized systems appear to be considered reliable [21–23]. However, the process of manually identifying cephalometric landmarks on cephalograms requires a lot of time and has possibility of errors regardless of the experience of the clinician. Since the first study on automatic identification of cephalometric landmarks by Levy-Mandel et al. [24] in 1986, several researchers have tried to automate landmark identification using knowledge-based techniques or image matching methods and learning systems. However, only a few clinical studies have been conducted on automatic landmark identification [25–28].

The program used in this study was Ceph-X. The program is based on the machine learning; automatic landmark localization algorithm is based on convolutional neural network. The program requires the confirmation of landmark position before calculating measurements. Full automation of all steps is challenging due to overlaying structures and inhomogeneous intensity values in the cephalometric radiographs. Thus, calculating measurements process may not be performed by AI. This study is conducted to provide a clear picture about the possibility of replacing the traditional cephalometric process with the digital one. The study focused mainly to evaluate its usability for cephalometric analysis and measurements using automated program.

A previous study reported that this system shows an accuracy of 96.6% when compared with manual cephalometric approaches, with an acceptable variation of less than approximately 0.5 mm and 1° [29]. Our results showed that three measurements, including the saddle angle, linear measurements of maxillary incisor to NA line and mandibular incisor to NB line exhibit statistically significant differences between the conventional and AI methods. The landmark identification of tooth structures can be affected by the surrounding superimposing anatomical structures, and clinicians also make this error. Particularly, identifying the mandibular incisor is difficult because it is generally located below the maxillary incisor due to overjet and overbite. Moreover, the widths of limits of agreement in the Bland-Altman plots were wider in dental measurements than those in the skeletal measurements. AI may have lower accuracy of performance in detecting tooth structures. The soft tissue measurements did not show any significant difference between the conventional and AI methods.

Based on the Bland-Altman plots, the measurements are in sufficiently good agreement. In the plots, the measurements that showed significant differences in the paired *t* test were within the limit of agreement (Figs. 2, 3, and 4). The wide limits of agreement in the Bland-Altman plots were defined clinically. Although there were statistically significant differences in some measurements and wide limits of agreement in the Bland-Altman plots between the two methods, the cephalometric analysis can be performed faster with the AI technique. In the present study, no manual adjustment after automatic landmark digitization was performed in order to exclusively evaluate the AI performance. With some manual adjustment made to landmark identification, the AI technique for cephalometric analysis may provide good performance. Considering that AI technologies will continue to improve in terms of the accuracy of measurement analysis with additional data and increasing use, the accuracy of cephalometric analysis based on AI techniques applied to clinical orthodontics will only further improve. Previous study by Hwang et al. [15] using recently proposed deep-learning method has reported that the mean error in landmark detection between AI and human was 1.46 ± 2.97 mm. In the present study, the mean error in all cephalometric measurements between conventional method and AI was 0.6 ± 3.1 mm. Although the errors in landmark identification cannot be compared directly with cephalometric measurements, the error using AI may be acceptable in clinics. In the study, the time needed for automatic tracing was within 5 s. In the conventional method, the mean time for tracing was about 6 min. Correcting lines requires lots of time. Considering this, automatic cephalometric analyses could help clinicians with manual adjustment.

The limitation of this study is that the sample size is smaller than that employed in previous studies on AI and machine learning technologies [30, 31]. In addition, one kind of radiographic machine was used to take cephalometric radiographs in the present study. Since the software used in this study is a commercially available cephalometric analysis program, it is believed that the performance of the software may be same with the images taken by various radiographic machines.

## Conclusion

With the limitation of this study, the results indicate that automatic cephalometric analyses based on convolutional neural network may offer clinically acceptable diagnostic performance. Careful consideration and additional manual adjustment are needed for dental measurements regarding tooth structures for higher accuracy and better performance.

## Data Availability

The data and materials obtained in this study belong to the authors, and are therefore available only upon request, after approval by the authors.

## Abbreviations

AI: Artificial intelligence
CAD/CAM: Computer-aided design/computer-aided manufacturing
